# Role of locomotor efference copy in vertebrate gaze stabilization

**DOI:** 10.3389/fncir.2022.1040070

**Published:** 2022-12-09

**Authors:** Hans Straka, François M. Lambert, John Simmers

**Affiliations:** ^1^Faculty of Biology, Ludwig-Maximilians-University Munich, Munich, Germany; ^2^Institut de Neurosciences Cognitives et Intégratives d’Aquitaine (INCIA), CNRS UMR 5287, Université de Bordeaux, Bordeaux, France

**Keywords:** locomotion, gaze stabilization, extraocular muscles, vestibular system, *Xenopus laevis*, spinal efference copies, metamorphosis

## Abstract

Vertebrate locomotion presents a major challenge for maintaining visual acuity due to head movements resulting from the intimate biomechanical coupling with the propulsive musculoskeletal system. Retinal image stabilization has been traditionally ascribed to the transformation of motion-related sensory feedback into counteracting ocular motor commands. However, extensive exploration of spontaneously active semi-intact and isolated brain/spinal cord preparations of the amphibian *Xenopus laevis*, have revealed that efference copies (ECs) of the spinal motor program that generates axial- or limb-based propulsion directly drive compensatory eye movements. During fictive locomotion in larvae, ascending ECs from rostral spinal central pattern generating (CPG) circuitry are relayed through a defined ascending pathway to the mid- and hindbrain ocular motor nuclei to produce conjugate eye rotations during tail-based undulatory swimming in the intact animal. In post-metamorphic adult frogs, this spinal rhythmic command switches to a bilaterally-synchronous burst pattern that is appropriate for generating convergent eye movements required for maintaining image stability during limb kick-based rectilinear forward propulsion. The transition between these two fundamentally different coupling patterns is underpinned by the emergence of altered trajectories in spino-ocular motor coupling pathways that occur gradually during metamorphosis, providing a goal-specific, morpho-functional plasticity that ensures retinal image stability irrespective of locomotor mode. Although the functional impact of predictive ECs produced by the locomotory CPG matches the spatio-temporal specificity of reactive sensory-motor responses, rather than contributing additively to image stabilization, horizontal vestibulo-ocular reflexes (VORs) are selectively suppressed during intense locomotor CPG activity. This is achieved at least in part by an EC-mediated attenuation of mechano-electrical encoding at the vestibular sensory periphery. Thus, locomotor ECs and their potential suppressive impact on vestibular sensory-motor processing, both of which have now been reported in other vertebrates including humans, appear to play an important role in the maintenance of stable vision during active body displacements.

## Introduction

**FIGURE 1:**
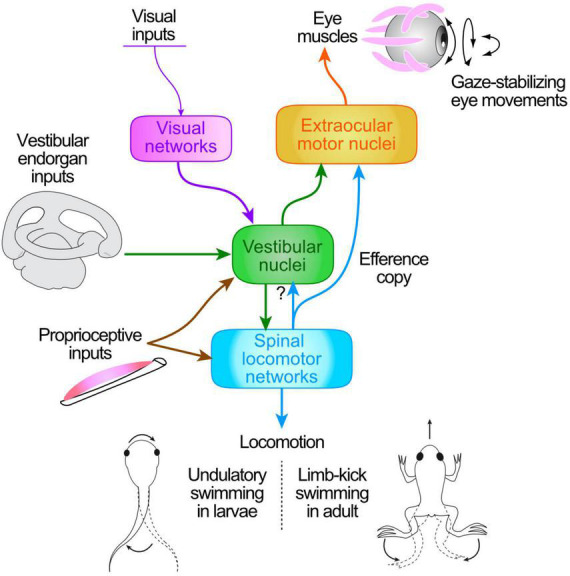
Schematic of central pathways responsible for the transformation of head/body motion-related sensory signals (from visual motion, vestibular endorgans, neck proprioceptors) and ascending motor efference copies (ECs) from spinal locomotor CPG networks into ocular motor commands for gaze stabilization during tail undulatory swimming in *Xenopus* larvae **(bottom left)** and limb-kicking propulsion in adults **(bottom right)**.

Gaze stabilization during both self-generated and passive motion is essential for constantly maintaining retinal image acuity as a prerequisite for stable perception of the visual world (Straka and Dieringer, 2004). During passive displacements of the head/body, gaze-stabilizing reactions are produced by the transformation of motion-related sensory signals into motor commands that drive counteracting movements of the eyes and neck (Angelaki and Cullen, 2008). The relevant sensory signals originate from peripheral vestibular sense organs, motion-sensitive retinal ganglion cells and neck proprioceptors (Figure 1), which together detect rotational and translational body motion in space as well as changes in head orientation with respect to the body and to gravity (Cullen, 2004, 2012; Angelaki and Cullen, 2008). Following central integration, the output signals are conveyed directly and/or indirectly to extraocular and neck motoneurons that in turn drive reactive gaze-stabilizing muscle contractions (Cullen, 2016). The integrative principles underlying central sensory-motor processing are best illustrated by vestibulo-ocular reflexes (VORs), which have long been considered as the canonical and dominating neural mechanism in all vertebrates for stabilizing gaze during head motion in space (Straka and Dieringer, 2004; Angelaki and Cullen, 2008).

While the sensory-motor transformations subserving gaze stabilization are well documented for passive head/body motion, the processes operating during self-generated movements have remained more elusive. Over the last decade, however, evidence has accumulated that intrinsic copies of the actual motor commands responsible for an animal’s propulsive axial or limb movements may also provide a source of neuronal signals for retinal image stabilization (Figure 1). Due to the spatio-temporal predictability of head movements and resultant visual perturbations that accompany the generally stereotyped expression of locomotor activity (Chagnaud et al., 2012), efference copies (ECs) of the underlying rhythmic motor output constitute convenient and reliable signals for predicting the sensory consequences of actual locomotor movements (Straka et al., 2018). In this case, these intrinsic feed-forward signals (Sperry, 1950; von Holst and Mittelstaedt, 1950) that arise from spinal central pattern generator (CPG) circuits (see Stehouwer, 1987; Combes et al., 2004, 2008) rather than reflex-based sensory-motor transformations, would be responsible for ensuring retinal image stability during locomotion (Lambert et al., 2012). Providing that appropriate neuronal circuitry is available to couple the spinal cord with brainstem ocular motor targets, such spinal ECs constitute faithful predictive representations of locomotor motion dynamics, and thereby are able to directly offset cyclic, self-movement-induced visual perturbations and drive compensatory eye adjustments (Chagnaud et al., 2012).

**FIGURE 2:**
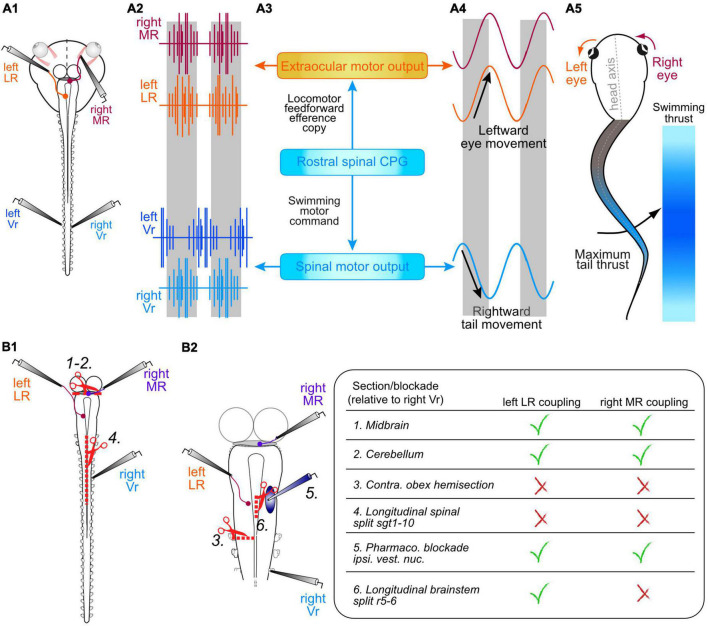
Semi-intact preparation of *Xenopus* larvae for studying the influence of spinal motor network ECs in gaze stabilization during fictive locomotion. **(A1–5)** Schematic of a head-brainstem-spinal cord preparation **(A1)** depicting recordings **(A2)** of a left and right spinal ventral root (Vr, blue traces), a synergistic left lateral rectus (LR, orange trace) and a right medial rectus (MR, red trace) extraocular motor nerve; direct spino-ocular motor connectivity **(A3)** conveys a copy of swimming motor output from the spinal CPG circuitry to drive compensatory conjugate eye movements during head movements resulting from propulsive bending **(A4)**, mainly of the mid-tail region [indicated by color intensity grading in panel **(A5)**]. **(B1,2)** Schematics of the isolated central nervous system **(B1)** illustrating various surgical lesions and drug application interventions that allowed identifying the ascending pathway from the spinal CPG circuitry to bilateral synergistic sets of LR and MR extraocular motoneurons; 1, 2, disconnection of the midbrain [1 in panel **(B1)**] and cerebellum [2 in panel **(B1)**]; 3, hemisection of the brainstem/spinal cord at the level of the obex on the side ipsilateral to a recorded (left) LR nerve **(B2)** whose burst activity occurred in phase with Vr bursts on the opposite (right) cord side; 4, longitudinal midline split throughout the first 10 spinal segments **(B1)**; 5, injection of glutamatergic transmitter antagonists (CNQX + KYNA) into the vestibular nucleus to block central processing of sensory endorgan signals **(B2)**; 6, midline incision at the level of the abducens nucleus in rhombomere 5 **(B2)**. These interventions and their respective effects on spino-ocular motor coupling to the synergistic left LR and right MR nerves are summarized in the table at right.

Although conceptually plausible, the actual implementation of such a gaze-stabilizing mechanism is experimentally challenging to demonstrate in intact animals, since during locomotor behavior, spinal feed-forward signals are difficult to distinguish from muscle proprioceptive feedback signals arising from the contractions of axial and limb muscles. Thus, obtaining unequivocal evidence for locomotor EC-driven gaze stabilization requires an experimental approach and tractable animal model that allow dissociating the generation of spinal locomotor output commands from the resultant production of propulsive muscle contractions and associated sensory feedback signals. This condition has been achieved through the development of semi-intact preparations of larval and young adult *Xenopus laevis*, consisting of the head—including inner ears, eyes and eye muscles—along with the still-attached and isolated spinal cord (Figure 2A1; Straka and Simmers, 2012). Such preparations spontaneously produce episodes of swimming motor output activity—so-called “fictive locomotion”—consisting of rhythmic impulse bursts in spinal motor roots (Figure 2A2) that would normally drive axial and/or limb muscle contractions and propulsive body motion in the intact animal (Figures 2A3–5; Combes et al., 2004). Consequently, the absence of any active movement and resultant vestibular, visual and proprioceptive signaling in these reduced preparations allows a thorough testing of whether intrinsic ECs of the locomotor output are able to directly drive retinal image-stabilizing eye movements without the involvement of motion-related sensory feedback.

Here, we review evidence for the involvement of this novel mechanism during rhythmic undulatory and limb-based swimming in larval and juvenile *Xenopus*, respectively, as well as its ability to express locomotor mode-dependent plasticity during intermediate stages of metamorphic development as the larva transforms into an adult frog. Moreover, we present evidence that spatio-temporally specific interactions of these intrinsic feed-forward signals with motion-generated feedback signals (such as those that initiate VORs) may provide a functional blueprint that extends to other vertebrates, including humans.

## Evidence for efference copy-driven eye movements during locomotion

During tail-based swimming in larval *Xenopus*, which occurs mostly in the horizontal plane, both eyes oscillate in conjugation during left-right head excursions resulting from undulatory tail movements (Figures 2A4,5; Lambert et al., 2012, 2020). This self-motion related pattern, produced by alternate contractions of synergistic pairs of lateral rectus (LR) and medial rectus (MR) eye muscles, corresponds to the oppositely-directed eye movements generated by VORs during passive oscillatory head motion (Straka and Dieringer, 2004). However, the ocular motor commands occurring during active swimming were found to derive from the spinal CPG circuitry itself, as demonstrated in semi-intact and further reduced (isolated brainstem/spinal cord) *in vitro* preparations by the functional coupling of ocular and spinal motor activity during spontaneous fictive locomotion (Combes et al., 2008; Lambert et al., 2012). During such locomotor episodes, evidenced by left/right alternating spike bursts recorded in spinal ventral roots (Vrs), a robust burst discharge timed to the Vr activity also occurs in the LR and MR extraocular motor nerves (Figure 2A2). This phase-coupled extraocular motor activity expressed by preparations devoid of any tail motion or visuo-vestibular sensory feedback, therefore provided the initial compelling evidence that locomotor ECs access the ocular motor system (Figure 2A3) during actual swimming behavior in the intact animal and therefore likely contribute to gaze stabilization (Combes et al., 2008; Lambert et al., 2012, 2020).

## Specificity of locomotor efference copy signaling in stabilizing gaze

The spino-ocular motor coupling that utilizes ECs during fictive swimming in larval *Xenopus* was found to be as equally effective in producing gaze-stabilizing eye movements as reflexive sensory-motor transformations during passive head/body motion. This equivalence is due to the identical spatio-temporal specificity of the neural processes associated with the spino-ocular motor coupling.

### Temporal specificity of coupling

The functional adequacy of spinal locomotor ECs in driving eye movements was first evidenced by the relative timing of the discharge of bilateral horizontal extraocular motor nerves and spinal Vrs monitored in semi-isolated preparations during spontaneous episodes of fictive swimming (Combes et al., 2008; Lambert et al., 2012). Specifically, such recordings revealed that impulse bursts in an LR nerve on a given side occur in phase with rhythmic Vr discharge on the opposite spinal cord side, whereas bursts in each MR nerve are phase-locked with Vr bursts on the same cord side. This strict temporal relationship is consistent with the production of conjugate horizontal rotations of the eyes that are oppositely directed to head undulations resulting from active tail motion in the intact animal. The coupling pattern is therefore functionally appropriate for producing gaze-stabilizing eye movements during actual swimming behavior. Moreover, an earlier biomechanical study of larval morphology together with a kinematic analysis of its swimming movements revealed that the mid-caudal part of the tail, where the dorsal and ventral fins are largest, corresponds to the most effective propulsive region, producing maximal hydrodynamic thrust per swim cycle (Wassersug and von Seckendorf Hoff, 1985). Correspondingly, the delay in coupling between ocular and spinal motor bursts was found to be constantly adapted to ensure conjugate eye movements that precisely counteract the undulations of the mid-caudal tail region, corresponding to the 15th–20th myotomal segments (Figures 2A4,5; Bacqué-Cazenave et al., 2018).

### Activity-dependence of coupling

The strength and frequency of swimming by *Xenopus* tadpoles can vary spontaneously across a broad range, with resultant alterations in accompanying head oscillation amplitudes and frequencies (Hänzi and Straka, 2017). Such variations in actual swimming performance are represented *in vitro* by differences in the intensity and frequency of spinal Vr bursts during fictive locomotor episodes, as well as by the extent of Vr activation along the cord (Combes et al., 2004; Hänzi and Straka, 2017). During strong fictive swimming, typically all Vrs exhibit bilaterally-alternating bursts with a brief, rostro-caudally propagating segmental delay. Declining fictive swim strengths are evidenced by Vr burst activity occurring exclusively in more caudal spinal segments, in correspondence with tail undulations restricted to the most caudal regions during weak swimming *in vivo* (Combes et al., 2004). Although bursting persists in more caudal roots during low intensity fictive swimming, spino-ocular motor coupling disappears when the Vrs of the first 10 spinal segments fall silent (Lambert et al., 2012; Bacqué-Cazenave et al., 2018). This region-specific coupling therefore corresponds to the requirement for compensatory eye movements only when the rostral axial musculature is activated and produces substantial horizontal head deviations during strong swimming (Bacqué-Cazenave et al., 2018). Such an activity-dependent profile thus indicates that spino-ocular motor coupling varies with the rostro-caudal recruitment of the segmental CPG circuitry and the dynamics of the resulting tail/head undulations.

### Spatial specificity of coupling

Based on the anatomical organization and pulling directions of epaxial and hypaxial muscles along the trunk and tail, undulatory swimming in *Xenopus* tadpoles occurs mainly in the horizontal plane (Wassersug and von Seckendorf Hoff, 1985; Combes et al., 2004; Azizi et al., 2007), thereby necessitating compensatory eye movements preferentially in this spatial plane. Systematic recording of the extraocular motor nerves to all six eye muscles in semi-isolated preparations indicated that phase-coupled rhythmic burst discharge during fictive swimming is indeed restricted to MR and LR motoneurons that control horizontal eye movements, while activity in motor nerves to the four vertical and oblique eye muscles remains unmodulated by the cyclic locomotor bursting (Lambert et al., 2012). This finding therefore indicates that the transmission of spinal ECs employs a target-selective connectivity that is limited to the spatially-specific prediction of horizontal motion during undulatory swimming.

## Spinal origin and ascending transmission of locomotor efference copies

### Exclusive spinal origin of gaze-stabilizing ocular motor commands during locomotion

A defining feature of the functional association between frog locomotion and gaze-stabilizing eye movements is that the ocular motor drive arises from signals in the spinal cord, at variance with all other known premotor sources responsible for eye movement control (Horn and Straka, 2021). The uniquely spinal origin of the ECs that drive rhythmic LR and MR nerve bursting was established by the surgical removal of supraspinal areas that are typically involved in locomotor control, such as the midbrain locomotor center (Saitoh et al., 2007) or the cerebellum (Arshavsky et al., 1984; Armstrong et al., 1997) in isolated brainstem/spinal cord preparations (Figure 2B1, lesion 1, 2). The finding that spino-ocular motor coupling during fictive swimming persists with unaltered magnitude and timing after the disconnection of either structure (Lambert et al., 2012), along with a maintained similarity between rostral spinal Vr and LR/MR nerve bursts, strongly indicates that the EC drive derives solely from CPG circuitry in the rostral spinal cord.

### Trajectory of ascending spino-ocular motor pathways

The axonal pathways that mediate the spino-ocular motor coupling were identified by various anatomically restricted lesions that would sever potential pathway trajectories between the spinal cord and the target extraocular motor nuclei in the mid- and hindbrain (Lambert et al., 2012). A hemi-section of the hindbrain/spinal cord at the level of the obex suppressed CPG-driven bursts in the LR motor nerve on the side ipsilateral to the lesion (Figure 2B2, lesion 3), which were otherwise timed to Vr motor bursts on the opposite cord side. In contrast, the magnitude and timing of ongoing rhythmic bursts in the LR nerve on the contralesional side remained unaffected. In addition, spino-LR motor nerve coupling persisted after a midline separation of the entire hindbrain that extended until the obex (not illustrated), whereas a longitudinal division of the spinal cord that descended from the obex until segment 10 abolished any spinal CPG-driven bursting in LR motoneurons on both sides (Figure 2B1, lesion 4). Since LR nerve bursts on one side are phase-coupled with the Vr locomotor burst rhythm on the opposite side, these lesion experiments confirmed that the locomotor EC for each left/right alternating phase originates contralaterally in the rostral cord region and is conveyed by neurons with ascending axons that project to the brainstem *via* a trajectory that crosses the midline at a level below the obex.

Upon reaching the brainstem, a seemingly convenient next step in routing EC information to LR motoneurons could involve a relay *via* the central vestibular nuclei, where the spinal signals could be integrated with incoming motion-related vestibular sensory inputs. Such signal transmission through the well-defined VOR circuitry (Straka and Dieringer, 2004) would also provide a suitable substrate for distributing EC activity to specific and functionally synergistic sets of extraocular motoneurons. This possibility was tested by exploiting the ability of the vestibular endorgans in semi-intact preparations of *Xenopus* tadpoles to detect and encode cyclic head motion when mounted on a turn-table motion stimulator while the reflex responses of LR motoneurons and the resultant eye movements are monitored concurrently (Straka and Simmers, 2012). A pharmacological blockade of glutamatergic inputs to second-order vestibular neurons following focal injection of AMPA and NMDA receptor antagonists into the vestibular nuclei (Figure 2B2, intervention 5; Cochran et al., 1987) during such imposed head rotation abolished the reflex activation of LR motoneuron but without affecting the spinal CPG drive to these neurons, either independently of, or conjointly with, the expression of fictive swimming (Lambert et al., 2012). This insensitivity of spino-ocular motor coupling to a glutamatergic synaptic blockade in the vestibular nuclei therefore indicates that the latter are in fact not implicated in relaying ascending locomotor EC signals from the spinal cord to horizontal extraocular motoneurons. But rather, it points to the involvement of either a different relaying center or a direct transmission pathway to LR motoneurons. In support of the latter possibility, electrical stimulation of the ventral funiculus evoked action potentials in the ipsilateral LR nerve with a very brief and constant delay, compatible with a direct monosynaptic connection (Lambert et al., 2012). The putatively single synapse interposed between the rostral spinal cord and LR motoneurons in the abducens nucleus was confirmed by neuroanatomical tract tracing from this nucleus into the spinal cord, which revealed a distinct subset of neurons with relatively small, segmentally-iterated somata located in the dorso-lateral marginal zone in each of the first 10 spinal segments. The axons of these neurons project ventrally beneath the central canal, cross the midline in the segment of origin and then ascend in ventro-medial fiber bundles to reach the abducens nucleus in the hindbrain (Lambert et al., 2012).

**FIGURE 3:**
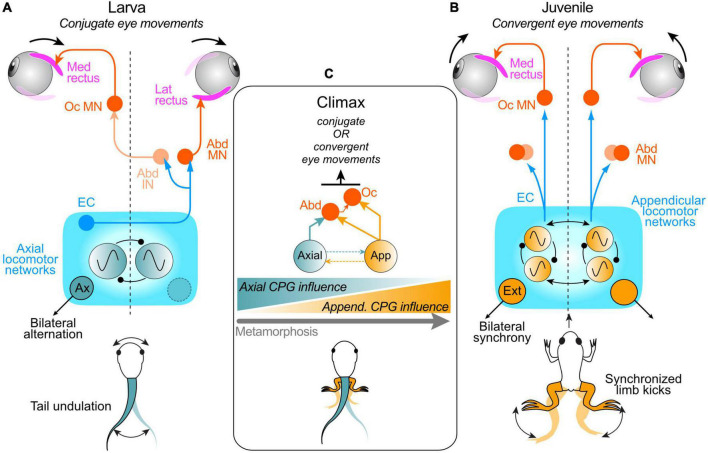
Neuronal pathways underlying spino-ocular motor coupling during swimming in pre- and post-metamorphic *Xenopus*. **(A)** Schematic depicting the spinal CPG for tail-based propulsion in larvae and pathway connections that convey spinal EC signals to the extraocular motor system; after crossing the spinal cord, ascending projections from the spinal axial CPG network directly drive abducens motoneurons (Abd MN) innervating the lateral rectus (LR) eye muscle on the opposite side and indirectly *via* abducens internuclear neurons (Abd IN) drive oculomotor motoneurons (Oc MN) innervating the synergistic medial rectus (MR) eye muscle on the same side; this spino-ocular connectivity produces conjugate eye movements during swimming. **(B)** Schematic depicting the spinal CPG for limb-based rectilinear forward propulsion in juvenile *Xenopus* and pathway connections with the extraocular motor system; ascending, uncrossed projections from the spinal appendicular locomotor network activate ipsilateral Abd MNs and Oc MNs to produce convergent eye movements during each hindlimb extensor-driven kick cycle. **(C)** During metamorphic climax, larval and adult CPG circuits co-exist and can be conjointly active, producing a combination of tail- and limb-based propulsion and corresponding EC signals that continuously elicit appropriate conjugate and convergent eye movements; the influence of the appendicular system on ocular motor control progressively increases as the impact of the axial system declines as limb-based locomotion emerges, co-exists with, and eventually replaces tail-based locomotion during metamorphosis (schematized at bottom).

The production of conjugate eye movements during swimming in larval *Xenopus* requires alternating spike bursting of LR motoneurons on one side timed with spike bursts of synergistic MR motoneurons in the oculomotor nucleus on the contralateral side (Lambert et al., 2008). Accordingly, since locomotor-related MR nerve bursting on a given side occurs in phase with ipsilateral spinal Vr bursts, the EC signals could reach the midbrain oculomotor nucleus directly *via* an ascending ipsilateral pathway that is separate from the cross-cord pathway to LR motoneurons in the abducens nucleus. Alternatively, the same copy signals reaching LR motoneurons in the abducens nucleus in rhombomere 5 could be conveyed onward to MR motoneurons on the opposite side by brainstem midline crossing axons of the abducens internuclear neuron (Abd IN) pathway that is known to be involved in producing conjugate eye movements (e.g., Highstein and Baker, 1978, and see below). A direct uncrossed ascending spino-oculomotor nuclear (MR) pathway was excluded, at least at larval stages (see below in the case of post-metamorphic frogs), by a hemi-section lesion at the level of the obex, which did not prevent rhythmic locomotor-timed bursting in MR motoneurons on the ipsi-lesional side (Lambert et al., 2012). On the other hand, a brainstem hemi-section rostral to the abducens nucleus in rhombomere 5 abolished MR nerve bursts on the side of the lesion, consistent with an interruption of Abd INs constituting the midline re-crossing EC signaling pathway from the abducens to the oculomotor nucleus. In addition, a localized longitudinal midline incision at the level of the abducens motor nucleus in rhombomere 5 (Figure 2B2, lesion 6) suppressed locomotor EC-driven MR nerve bursting on both sides, without affecting spino-LR motoneuronal coupling. Finally, a pharmacological blockade of glutamatergic synaptic transmission by a strictly unilateral injection of glutamatergic receptor antagonists into the abducens nucleus suppressed spinal CPG-driven bursting in both the LR nerve on the injected side and the contralateral synergistic MR nerve. In contrast, the locomotor-related burst rhythm in the antagonistic LR/MR nerve pair remained unaffected. Together, these findings confirmed that the re-crossing of locomotor EC signals to reach MR motoneurons in the midbrain indeed occurs through Abd INs (Figure 3A, left; Lambert et al., 2012), which in amphibians as in other vertebrates ensure conjugate eye movements during the horizontal angular VOR (e.g., Highstein and Baker, 1978; Delgado-Garcia et al., 1986; Graf et al., 1997; reviewed in Straka and Dieringer, 2004).

## Adaptive developmental plasticity of locomotor efference copy-driven eye movements

During *Xenopus* metamorphosis, the gradual transformation in body plan from a larva to an adult frog is accompanied by a switch in the animal’s locomotor strategy from tail-based undulatory movements to bilaterally-synchronous hindlimb kicking in the adult. This dramatic change in propulsive mechanism and movement patterns during metamorphosis requires a concurrent reconfiguration of the underlying spinal CPG circuitry, which in the adult frog drives rhythmically-alternating contractions of ipsilateral extensor and flexor muscles in bilateral synergy (Combes et al., 2004). A major consequence of this transformation is the complete alteration in body motion pattern and dynamics. The limb-based rectilinear forward motion of post-metamorphic frogs no longer requires conjugate left-right eye rotations for effective retinal image stabilization. Instead, as predicted from passive linear translational motion-induced eye movements (Straka and Dieringer, 2004), hindlimb extension-driven forward propulsion in adult frogs must be accompanied by convergent eye movements to ensure binocular focal plane stabilization as the animal swims toward a potential visual target.

### Spino-ocular motor coupling pattern in post-metamorphic frogs

Kinematic analyses of concurrent limb and eye motion during locomotor activity *in vivo* along with *in vitro* recordings and specific lesions of the central nervous system have established the pattern of spino-ocular motor coupling and underlying neural pathways in young adult *Xenopus* frogs (Figure 3B; von Uckermann et al., 2013, 2016). Simultaneous video imaging of limb and eye movements indicated that during cyclic hindlimb extension-driven forward propulsion, the two eyes do indeed rotate inwardly (convergence) instead of performing conjugate left-right oscillations as found in larvae (von Uckermann et al., 2013). Compatible with actual movements in the intact animal, recordings of extraocular and limb motor nerves in isolated froglet preparations during fictive swimming revealed a corresponding change in spinal EC influence on extraocular motor output (von Uckermann et al., 2013, 2016). In contrast to larval fictive swimming, where alternating bursts occur in antagonistic pairs of horizontal (LR, MR) extraocular motor nerves on the two sides, respectively, fictive limb kicking in adult frogs is accompanied by concurrent rhythmic bursting in the two MR nerves. These bilateral MR nerve bursts are strictly phase-coupled to bilaterally-synchronous hindlimb extensor nerve bursts, consistent with the *in vivo* production of convergent eye movements during linear forward accelerations produced by each limb kick cycle. It is noteworthy also that the temporal and magnitude relations between LR/MR nerve bursts and ongoing fictive swimming remained unaltered in isolated brainstem/spinal cord preparations after surgical ablation of the midbrain and cerebellum, thus indicating that, as found at larval stages, neither supraspinal structure participates in conveying the EC signals from the spinal cord to the extraocular motor nuclei.

### Functional organization of spino-ocular motor coupling in post-metamorphic frogs

The fundamental switch in coupling pattern between larval and adult frogs is accompanied by a corresponding change in the projection pattern of ascending spinal pathways, which after metamorphosis, originate from the lumbar segments responsible for hindlimb motor control (von Uckermann et al., 2013, 2016). In addition, the neural pathways mediating the spino-ocular motor coupling switch during metamorphosis from a crossed, to a strictly uncrossed trajectory to accommodate the requirement for a co-activation of bilateral extraocular motoneurons during synchronous extensions of the juvenile frog’s two hindlimbs (Figure 3B). In contrast to the push-pull operation of antagonistic bilateral pairs of MR and LR motoneurons during larval swimming, the alternating ocular con- and divergence during stepwise rectilinear forward propulsion in adults appears to result from a co-contraction of bilateral LR and MR muscles during convergent eye movements (von Uckermann et al., 2013). This co-activation might serve to fine-tune the much smaller disconjugate eye movements that are required and/or to maintain muscle tension during the propulsive phase of each swim cycle. The considerable adaptive plasticity during metamorphosis thus enables spinal CPG-driven ocular motor activity to match the changing spatio-temporal requirements for eye movements during propulsive self-motion. Moreover, the implementation of locomotor ECs for gaze stabilization during the adult frog’s limb-based locomotion reveals that this intrinsic mechanism is not restricted to the relatively simple left-right undulatory tail/body motion of swimming amphibian larvae or fish, but might also be relevant for gaze control during vertebrate bi- or quadrupedal locomotion in general (see below).

### Transition in spino-ocular motor coupling pathways during metamorphosis

The question arises as to how metamorphosing *Xenopus* copes with the transition between the different spino-ocular motor interactions yet maintains effective compensatory eye movements as one locomotor strategy with completely different head/body motion dynamics gradually emerges and supplants the other. This is particularly relevant during the period around metamorphic climax when swim episodes can arise from tail and/or limb-based mechanisms coexisting within the same animal (Combes et al., 2004). As a consequence of this bimodal capability, the resultant head motion profiles require compensatory eye movements that arise from accompanying ECs of the activity of both axial and limb CPG circuits in the spinal cord. Video monitoring of eye movements together with extraocular motor nerve and Vr recordings from semi-intact *Xenopus* preparations at mid-metamorphic stages enabled the switch in spinal EC control of ocular motor behavior to be determined (Figure 3C; von Uckermann et al., 2016). During spontaneous fictive swimming in such head-stationary preparations, eye movements remained spatio-temporally adapted to the expected head motion profiles and consequent visual disturbances that would derive from the two co-expressed modes of locomotion. This therefore indicates the involvement of underlying EC signals arising from co-existing spino-ocular motor coupling pathways associated with both axial locomotor circuitry and the newly emerging hindlimb CPG. Prior to metamorphic climax, when the developing limb circuitry remains subordinate to the axial CPG (Combes et al., 2004), the latter continues to provide the effective EC drive to the extraocular motor centers, as in pre-metamorphic larvae; correspondingly, eye movements are mainly conjugate. However, immediately after metamorphic climax, i.e., 2–4 days later, the balance of influence switches as the secondary appendicular system progressively becomes the predominant propulsive mechanism. Correspondingly, the spino-ocular motor influence becomes increasingly engaged in producing convergent eye movements as the primary axial locomotor system declines and eventually disappears with tail resorption (Figure 3C; von Uckermann et al., 2016). Therefore, during metamorphosis, spino-ocular motor coupling and EC control of eye movements are able to continuously satisfy the changing gaze-stabilizing requirements throughout the transfer of CPG dominance as one locomotor strategy emerges and replaces the other.

## Ontogeny of locomotor efference copies and their interaction with visuo-vestibular sensory signals

Whereas free swimming behavior starts around larval stage 37/38 (Kahn et al., 1982), eye movements elicited by spinal EC signals occur about 24 h later at stage 42, together with visual- and otolith organ-driven ocular motor responses (Lambert et al., 2008; Bacqué-Cazenave et al., 2022). Thus, from early larval life, spino-ocular motor coupling commands, in varying combinations with visuo-vestibular reflexes (also see below), are able to produce compensatory conjugate eye movements that are suitably adapted to subsequent developmental variations in locomotor behavior, with an ability to constantly adjust the gain and phase relationship of coupling, regardless of swimming intensity (Bacqué-Cazenave et al., 2022).

During swimming in *Xenopus* larvae, the comparable spatio-temporal properties of spinal CPG feed-forward influences and of feedback-derived horizontal angular VORs resulting from actual head movements initially suggested that the two signal components are combined to enhance the efficacy and/or precision of ocular motor performance. Such an additive relationship between predictive EC signals and reactive sensory-motor transformations was tested in semi-intact larval preparations with functional labyrinthine endorgans that allowed an activation of the horizontal semicircular canals with passive motion stimuli either independently of, or conjointly with, real or fictive swimming (Lambert et al., 2012; Bacqué-Cazenave et al., 2022). Horizontal head rotations without swimming activity caused a strong modulation of the ocular eccentricity, which was in phase with contraversive head movements and thus compatible with the expected ocular motor output produced by a horizontal angular VOR (Straka and Dieringer, 2004; Lambert et al., 2008).

**FIGURE 4:**
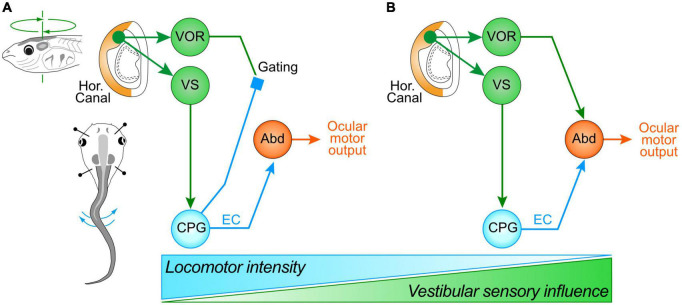
Differential swim intensity-dependent interactions between vestibular sensory signals and locomotor ECs. **(A)** Schematic depicting the gating of vestibulo-ocular signaling during intense swimming in older larvae or in rapidly-swimming young larvae, leading to ocular motor performance remaining coordinated with tail undulations exclusively through an ascending drive from spinal ECs; during less intense or slow swimming **(B)**, ocular motor performance results from an additive relationship between the predictive ECs and horizontal semicircular canal signals, with the latter now contributing significantly to compensatory eye motion; the two processing configurations gradually change their respective dominance depending on swimming strength and frequency (bottom schematic). Abd, abducens motoneurons; CPG, central pattern generator; VOR, vestibulo-ocular reflex; VS, vestibulo-spinal pathway.

During combined locomotor activity and passive head rotations, eye movements and underlying LR nerve bursting displayed different response patterns depending on the tail motion intensity. When swimming occurs at a relatively high frequency (>7 Hz), the ocular motor performance remains exclusively coordinated with the tail undulations, without any evident VOR contributions to eye motion (Lambert et al., 2012; Bacqué-Cazenave et al., 2022). The strictly unaltered profile of locomotor-related LR nerve bursts irrespective of the direction of imposed head motion indicates that horizontal semicircular canal signals, transmitted specifically through the vestibulo-ocular circuitry, are in fact suppressed by spinal ECs during concurrent fictive swimming (Figure 4A). However, when swim-related tail undulations occur at a relatively low frequency, the ocular motor performance was recently found to result from an additive relationship between the predictive EC drive and reactive horizontal semicircular canal signals, with a clear contribution of the latter to compensatory eye motion and ocular eccentricity (Figure 4B; Bacqué-Cazenave et al., 2022). This additive relationship was discernible by the appearance of two distinct frequency components (swim and head rotation frequencies) in eye motion spectrograms obtained by fast Fourier transformation during combined cyclic head rotation and low frequency swimming, whereas spectrograms during high frequency swimming lacked the sensory-derived component related to head rotation (Bacqué-Cazenave et al., 2022). The summation of spino-ocular motor commands and VOR responses therefore indicates that during less intense or slower rhythmic swimming behavior, EC signals are no longer capable of gating-out horizontal semicircular canal inputs. Furthermore, since swimming frequency decreases steadily during larval life as animals increase in size (Hänzi and Straka, 2017), the additive contribution of the horizontal VOR to visual image stabilization during rhythmic self-motion becomes more predominant with age (Bacqué-Cazenave et al., 2022).

**FIGURE 5:**
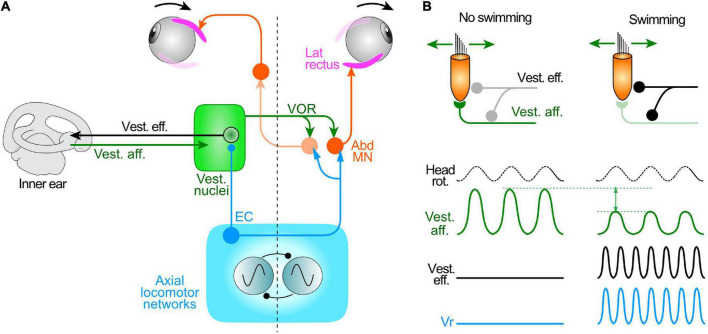
Attenuation of mechanosensory signal encoding in the inner ear during locomotor activity in larval *Xenopus*. **(A)** Summary schematic of the circuit elements mediating the vestibulo-ocular reflex (VOR), spino-ocular motor coupling and activation of inner ear vestibular efferent neurons (Vest. eff.) by locomotor ECs. **(B)** Influence of locomotor activity conveyed by Vest. eff. onto hair cells and vestibular afferent neurons (Vest. aff.); during swimming (right), the overall responsiveness of vest. aff. (magnitude of the sine wave) to imposed cyclic head rotation is substantially reduced compared to the condition without swimming (left). Abd MN, abducens motoneurons; Vr, spinal ventral root.

The suppression of vestibular afferent influences during high-intensity larval swimming might be spatially specific to inputs from the horizontal semicircular canals, or might represent a generalized filtering process that applies to all vestibular signals irrespective of their peripheral origin. To distinguish between these two possibilities, the influence of locomotor ECs on inputs from vestibular endorgans other than the horizontal semicircular canals was tested during passive motion around the longitudinal (roll) axis. In fact, an imposed left-right head roll motion caused a strong modulation of the LR nerve discharge that increased and decreased in strength during contra- and ipsiversive movements, respectively. The directionality of these evoked ocular motor responses and the low frequency (0.1 Hz) of the motion stimulus were consistent with the activation of vestibular signals predominantly arising from the gravitoinertial stimulation of utricular hair cells (Lambert et al., 2008). Significantly moreover, when locomotor activity and such passive roll motion stimulation occurred conjointly, the LR nerve firing pattern now expressed a combination of the motor response profiles produced separately under each experimental condition (for further details, see Figure 7 in Lambert et al., 2012).

The differential interactions between locomotor ECs and vestibular inputs from different endorgans indicates that the spinal feed-forward ECs are able to selectively impact on sensory feedback processing. Whereas horizontal angular VORs are employed in compensating for passive displacements of the head, during self-generated motion, these reflexes are specifically supplanted by spinal EC commands that assume the task of compensating for horizontal angular head rotation (Lambert et al., 2012; Straka and Chagnaud, 2017). Apart from the novel concept that plane-specific gaze stabilization in swimming *Xenopus* tadpoles can be conferred by locomotor ECs rather than by VOR-producing sensory-motor transformations, this mechanism also has hitherto unsuspected implications for issues ranging from the evolution of the horizontal semicircular canals to the maintenance of postural stability during locomotion in human patients with vestibular disorders (see below, and Lambert et al., 2012).

## Putative sites for vestibulo-ocular reflex gating

The suppressive impact of locomotor ECs on vestibular sensory processing (Lambert et al., 2012; Chagnaud et al., 2015) is commensurate with the classical role of such predictive signals in preempting the reactive engagement of movement sensing pathways, thereby providing an estimate of the sensory consequences of a behavioral action (Sperry, 1950; von Holst and Mittelstaedt, 1950). Although the sensory-motor transformations underlying VORs are generally relatively fast (Cullen, 2016), the timing and precision of the elicited compensatory eye movements might still be temporally inappropriate or even compromise the preemptive effects of spino-ocular motor coupling, especially during rapid larval swimming (Hänzi and Straka, 2017). Such a direct attenuating influence of spinal CPG activity on reflex pathways has been demonstrated for limb proprioceptive signaling in mammalian locomotory systems (for review, see McCrea, 2001), including humans (Dietz, 2003), and might in fact represent a more general phenomenon applicable to other sensory modalities not involved in controlling locomotion *per se*.

Thus far, neither the neural site(s) at which the suppression of spatially-specific signals in the VOR circuitry occurs, nor the underlying neurophysiological mechanism(s), have been fully established. Such an understanding requires identifying the anatomical pathways that convey the intrinsic CPG-derived signals and the suppressive processes operating at single or multiple synaptic levels. In principle, establishing these underlying features benefits from the simple organization of the VOR circuitry, which is comprised of a three-neuronal reflex arc for integrating angular and translational/gravitoinertial sensory signals (Lorente de Nó, 1933; Szentágothai, 1943, 1950; McCrea et al., 1980; Baker et al., 1981; Robinson, 1982; Baker, 1998; Fritzsch, 1998). This short-latency reflex pathway thus limits the potential sites for a functional interaction between spinal ECs and self-generated (reafferent) sensory signals to few potential neuronal *loci* that include the vestibular sensory periphery, central vestibular nuclei and extraocular motor nuclei themselves. While an EC influence on peripheral and central vestibular neuronal targets is plausible, a suppressive effect at the level of the extraocular motoneurons seems less likely, given the observed summating capability of sensory feedback with EC signals during concurrent locomotion and roll motion stimulation, as described above. Although a cancelation of vestibular signals at the motoneuronal level cannot be excluded, this situation would require a spatially-specific inhibitory action of spinal ECs at certain synapses of vestibulo-ocular neurons onto extraocular motoneurons. More likely, sites for an EC influence on vestibular processing are at earlier synaptic stages along the hierarchical VOR arc, namely pre- or post-synaptically at the first order (afferent fiber-vestibular projection neuron) synapse in the vestibular nuclei, or peripherally, at the synaptic connection between hair cells and vestibular nerve afferent fibers in the inner ear endorgans themselves (Straka and Dieringer, 2004).

### Locomotor efference copy influences on peripheral sensory encoding

Hair cells in the endorgans of the vertebrate inner ear receive both afferent and pre/postsynaptic efferent innervation (Figure 5A), although the role played by the latter during behavior has remained enigmatic (e.g., Holt, 2020; Mathews et al., 2020). Upon electrical stimulation, vestibular efferent neurons modulate the efficacy of hair cell-afferent fiber synaptic transmission (Boyle et al., 2009; see Holt, 2020) and thus offer a potential pathway substrate for the attenuation of mechano-electrical transduction and vestibular motion encoding during locomotor movement, on the condition that they themselves are activated centrally by spinal EC signals. Using semi-intact preparations of larval *Xenopus*, such an efferent neuron activation was indeed revealed by multi-unit recordings from the central severed ends of the anterior and posterior branches of a vestibular (VIIIth cranial) nerve (Chagnaud et al., 2015). During episodes of fictive swimming, both branches displayed a burst discharge that was phase-locked to rhythmic bursts in ipsilateral spinal Vrs. Even though vestibular afferent axons considerably outnumber their efferent counterparts (Birinyi et al., 2001; see Holt, 2020), that this rhythmic bursting was occurring in efferent pathways was confirmed by simultaneous recordings from both peripheral and central ends of a cut vestibular nerve, which showed the dissociated activities of vestibular afferent and efferent axons, respectively (Chagnaud et al., 2015).

The proportion of efferent neurons that are activated during locomotion was assessed by somatic calcium imaging during episodes of fictive swimming monitored by spinal Vr recordings. During swim episodes, Ca^2+^ transients were recorded in practically all efferent neuronal somata (of which there are 10–15 per side located in rhombomere 4), with onsets and durations that were strictly correlated with those of the accompanying spinal Vr bursting (Chagnaud et al., 2015). Furthermore, the similarity in Ca^2+^ response dynamics of different ENs during a given swim episode suggested a common underlying synaptic drive. Therefore, given the distributed peripheral projections of individual efferent neurons to multiple endorgans, it is probable that the entire vestibular efferent neuronal population participates in co-transmitting a copy of spinal EC activity to the inner ear during swimming. Moreover, the strong linear correlation between concurrent spinal Vr bursts and the generally bilateral (biphasic) burst discharge recorded in vestibular nerve branches indicated that the latter activity precisely encodes swim episode duration and any changes in Vr burst cycle frequency and intensity. This faithful coupling thereby ensures that the major parameters of propulsive motor commands are conveyed to the sensory periphery.

The impact of locomotor-related efferent neuron bursting on vestibular sensory encoding of head/body motion was evaluated in semi-intact larval *Xenopus* preparations with intact vestibular endorgans and preserved peripheral and central nerve connections (Figures 5A,B). Vestibular nerve afferent fibers were recorded *en passant* during rotational stimuli applied in different spatial planes alone, and during the occurrence of spontaneous fictive swimming (Chagnaud et al., 2015). In the absence of rhythmic locomotor activity, sinusoidal roll motion or left-right head rotations caused a cyclic discharge modulation in different sets of recorded vestibular afferent fibers (Figure 5B, left). However, during episodes of fictive swimming, EC activity conveyed by efferent neurons had a disparate influence on the spontaneous discharge and motion-induced modulation of vestibular afferent firing. Similar to the previously reported discrepant effects of direct electrical stimulation on efferent neuron spike discharge (Rossi et al., 1980; Myers et al., 1997), the mean firing rates of individual afferent fibers at rest and during passive horizontal motion either decreased or increased, or in a few cases remained unaffected during fictive locomotion (Chagnaud et al., 2015). However, despite this diversity of spinal CPG influences on the afferent population, the average magnitude of the motion-induced peak-to-peak discharge modulation of all recorded afferents was consistently reduced during fictive swimming by almost 50% compared to control periods when swimming was absent during passive motion (Figure 5B, right; compare with left).

The functional significance of the differential effects of efferent neuronal activity on the spontaneous firing of vestibular afferents and their sensitivity to motion stimuli might be related to the push-pull operation of the vestibular system itself. Here, the encoding of immobility relies on bilaterally-balanced population resting discharge rates throughout the afferent neuronal assemblage (Chagnaud et al., 2017). Accordingly, any asymmetrical deviation from the resting rate level between the two sides is interpreted centrally as the occurrence of head motion. Thus, maintaining bilaterally-balanced steady state firing rates of vestibular afferents, as a population, by averaging out the opposing effects of locomotor EC signals, would in turn ensure equilibrated resting activity within the central vestibular circuitry (Chagnaud et al., 2017). The overall decrease in the encoding of head/body movement mediated by ECs during locomotion is likely to be achieved at the synapses of individual efferent neurons with specific populations of hair cells and afferent fibers that innervate them, respectively diminishing the efficacy of (presynaptic) transmitter release and the gain of postsynaptic responsiveness (Holt, 2020). In agreement with proposals from a previous study on toadfish (Highstein and Baker, 1985), the EC influence might be channeled preferentially to specific sets of phasic afferent neurons and hair cells (Dlugaiczyk et al., 2019) that are capable of encoding the rapid movement dynamics associated with undulatory swimming. Consequently, whereas the impact of locomotor ECs on vestibular sensory encoding involves a diverse, yet overall maintained population resting discharge, stimulus encoding during actual self-motion becomes considerably attenuated.

### Locomotor efference copy influences on central sensory-motor transformations

Another potential site for a suppressive action of locomotor ECs on sensory-motor processing resides within the central circuitry of the vestibular nuclei (Straka and Dieringer, 2004). Since vestibular neurons form spatially-specific subsets involved in the three-dimensional transformations of head motion, any selective gating influences at this level could be readily targeted to relevant vestibular subpopulations (Straka et al., 2002). Indeed, EC-mediated sensory gating has been reported in monkeys during active and passive horizontal head motion (Roy and Cullen, 2004; Medrea and Cullen, 2013). Since vestibular nerve afferents similarly encode both actively- and passively-induced head movements (Sadeghi et al., 2007; Jamali et al., 2009), the observed cancelation, at least for voluntary self-motion-related vestibular signals such as during head orientation movements, must result from neuronal computations within the central vestibular nucleus itself (see Straka et al., 2016). A likely mechanism by which locomotor ECs could suppress, or at least considerably attenuate, central vestibular signaling is by a direct inhibition of semicircular canal-derived inputs through local glycinergic and/or GABAergic inhibitory circuits (Minor and Goldberg, 1991; Straka et al., 1997). While such local circuits effectively control the synaptic weighting of vestibular nerve afferent inputs by predominantly reducing phasic semicircular canal and otolithic inputs in central vestibular neurons (Straka and Dieringer, 2000; Biesdorf et al., 2008; Pfanzelt et al., 2008), their involvement in EC-mediated cancelation of vestibular sensory inputs remains to be demonstrated (also see below).

Although the selective suppression of ocular motor output in tadpoles during horizontal, but not vertical, head rotation is based on a spatial specificity, likely related to the plane of head undulations during axial swimming, an alternative explanation might involve a difference in the dynamic spectrum of activated vestibular afferent units during motion in the two rotational planes. Roll motion stimulation in the vertical plane activates sensory elements in the vertical semicircular canals but is also a highly effective gravito-inertial stimulus for the utricle, thereby activating a large proportion of hair cells/afferent vestibular fibers with more tonic response dynamics (Straka et al., 2009). This is at variance with the situation during horizontal rotation that causes an activation of neuronal elements with mostly higher response dynamics. Assuming that vestibular signal processing occurs in frequency-tuned channels (Straka et al., 2009; Elliott and Straka, 2022), it is possible that a suppressive influence of locomotor ECs predominantly targets those pathways that convey VOR signals with rapid dynamics, such as during swimming (Lambert et al., 2020). Given the preferential influence of local inhibitory vestibular circuits in gating phasic sensory signals (Straka and Dieringer, 2000), the same network components could also be accessed by spinal ascending pathways that relay locomotor ECs, thereby selectively attenuating fast dynamic vestibular sensory inputs during concurrent self- and passive body motion.

An involvement of cerebellar and local vestibular circuits in the gating of body motion-related sensory signals during locomotion would not be unexpected given previous knowledge of their roles in the control of gaze stabilization (Straka and Dieringer, 2004; Angelaki and Cullen, 2008). In contrast, mechanisms that regulate afferent sensory inflow to other regions of the central nervous system are more difficult to attribute to EC influences on vestibular sensory processing. In the spinal cord, the magnitude of proprioceptive afferent input resulting from movement is governed by a cellular mechanism involving primary afferent depolarization (PAD) of dorsal root afferents (Rudomin and Schmidt, 1999; Rudomin, 2009). This depolarization of muscle, cutaneous and articular afferent fibers is mediated by GABAergic interneurons that reduce sensory signal inflow to the spinal cord at the presynaptic afferent level (Rudomin, 2009). While there is so far no evidence for a similar presynaptic mechanism operating in the vestibular nucleus, PAD would nonetheless provide a highly suitable way to block the discharge of specific sets of vestibular nerve afferents by effectively shunting motion-related sensory signals before transmission to central vestibular neurons. Accordingly, locomotor ECs could activate GABAergic interneurons that cause both a postsynaptic inhibition of central neurons and a presynaptic depolarization of inner ear afferents, thereby selectively attenuating vestibular sensory inputs evoked by passive head motion. While PAD plays a special role in controlling motor performance, sensory processing, and coherence between programmed and executed limb movements (Rudomin, 2009), a comparable mechanism might also govern the interaction between vertebrate locomotion, motion sensing and gaze stabilization.

## Evolution and ubiquity of locomotor efference copy-driven gaze stabilization

In an evolutionary context, locomotor EC-driven eye movements might constitute a vestige of an ancestral mechanism, appearing in early vertebrates before rotational motion-encoding semicircular canals had appeared (Janvier, 2008). Although certain phylogenetic aspects remain under debate, the relatively late arrival of horizontal semicircular canals in jaw-bearing vertebrates (Retzius, 1881; Graf, 1988a,b; Beisel et al., 2005; Higuchi et al., 2019) occurred long after undulatory tail-based swimming and resultant horizontal rotations of a head region with motion sensors had emerged in aquatic tadpole-like chordate ancestors (Wada, 1998). By effectively providing a predictive correlate of horizontal angular motion, CPG-driven signaling of left-right body/head undulations could have been exclusively responsible for stabilizing retinal images in these early chordates (Wada, 1998). In this scheme, furthermore, the relatively late evolutionary appearance of horizontal semicircular canals through the recruitment of the ancestral gnathostome Otx gene (Fritzsch et al., 2001) might simply have been due to a lack of sufficient evolutionary pressure for acquiring an inner ear organ that specifically senses and encodes angular motion of the head in the horizontal plane (Chagnaud et al., 2012). The appearance of a more complex morphology would have enabled the semicircular canal sensory system to encode more efficiently a larger spectrum of head motion during evolution, particularly in aerial vertebrates (mammals and birds; Lambert and Bacqué-Cazenave, 2020). Improvements in locomotor performance and associated morphological inventions, such as flexible necks that reduced the predictability of locomotor-derived head movements (Chagnaud et al., 2012) were also likely to be evolutionary innovations that promoted the appearance of specific 3D movement-detecting sense organs. Concomitantly, these in turn might have changed the way in which locomotor EC signaling is employed in gaze stabilization.

While the functional processes and underlying neuronal pathways by which locomotor ECs can stabilize gaze have been unequivocally demonstrated in the amphibian *Xenopus laevis*, considerably less is known for such a role in other vertebrates. In lamprey, an extant jawless vertebrate species, the use of semi-intact, head-immobilized preparations after optic nerve transection and labyrinth ablation has very recently provided direct evidence that spinal CPG-derived ECs contribute to compensatory eye movements, coordinated with swimming undulations in the horizontal plane (Wibble et al., 2022). However, despite the lack of amenable experimental models and approaches, indications for a still wider involvement of locomotor ECs in stabilizing gaze and controlling vestibular sensory processing has emerged over the past decade. In guinea pigs, compensatory eye movements during self- generated head or body movements were found to be anticipatory, to occur independently of intact vestibular sensing and were likely to be produced through intrinsic feed-forward motor ECs that are able to predict the sensory consequences of the active head motion (Shanidze et al., 2010). Further supportive evidence for predictive signaling in mammals has derived from clinical studies on vestibular pathologies and the resulting deficits in gaze and posture (Brandt et al., 1999, 2001; Anson et al., 2017). The remarkable plasticity of the gaze and posture control systems involved in the amelioration of these deficits has been found to include an enhanced contribution of motor EC-driven (Sadeghi et al., 2010) or pre-programmed eye movements both in humans (e.g., Kasai and Zee, 1978) and animals such as guinea pigs (Haggerty and King, 2018). In addition, in both a dog that suffered from a unilateral vestibulopathy (Brandt et al., 1999) and human patients with similar unilateral vestibular deficits (Brandt et al., 2001), resultant postural instabilities were found to be significantly diminished during running as compared to walking. In assuming that stable locomotor movements in animals and humans alike are produced by the rhythmic activity of CPG circuitry in the spinal cord (Dietz, 2003), then ECs of this activity would presumably be stronger and most effective during the faster, more stereotyped and highly autonomous self-action of running (Dietrich and Wuehr, 2019a,b; Dietrich et al., 2020, 2022). Accordingly, the accompanying improvement in dynamic postural stability in both pathological situations was proposed to arise from a locomotor EC-derived reduction in bilaterally-asymmetric, and therefore perturbing, vestibular inputs, analogous to the situation in *Xenopus*. This idea has been further supported by experimental galvanic stimulation of bilateral inner ear endorgans in human patients, which caused smaller trajectory deviations during running than walking (Jahn et al., 2003), again compatible with an activity-dependent, spinal CPG-derived gating of vestibular sensory-motor transformations (Dietrich and Wuehr, 2019a,b; Dietrich et al., 2020, 2022).

**FIGURE 6:**
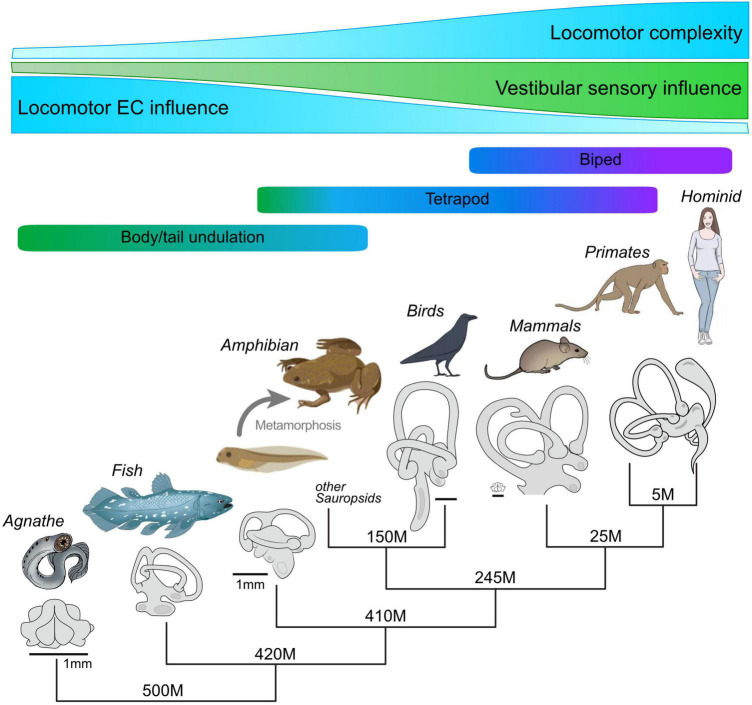
Hypothetical evolutionary development of locomotor complexity in vertebrates, the accompanying increase in morphological complexity and the role played by vestibular sensory signaling, and the resultant gradual decline in locomotor EC influence in the production of compensatory eye movements. Despite the increasing capability of inner ear endorgans for motion detection and sensory-motor transformations, locomotor EC signals appear to have maintained an important role in gaze stabilization, even during bipedal locomotion.

More direct evidence for locomotor EC-driven eye movements has recently been discovered in mice, where the existence of a comparable spino-ocular motor coupling has been established by multi-methodological approaches (França de Barros et al., 2021). Briefly, ocular motor activity remaining strictly phase-coupled to the rhythm of fictive locomotion was encountered in *ex vivo* brain-spinal cord preparations of neonatal mice with spatio-temporal characteristics reminiscent of those described in *Xenopus*. This ocular motoneuronal pattern complied with rhythmic eye movements, mostly in the horizontal plane, which occurred in phase with the forelimb gait pattern during treadmill-elicited locomotion in decerebrated animals. Moreover, the ascending locomotor signals were likely to derive from cervical cord neurons that connect directly with abducens motoneurons, similar to the situation found in amphibians (Lambert et al., 2012). Thus, spinal locomotor ECs evidently constitute a relevant intrinsic feed-forward signal for ensuring gaze stability during locomotion, not only in aquatic amphibians and likely in lamprey, but also in other, terrestrial vertebrates. Furthermore, the common organizational features found in amphibians and mammals suggest that a suppressive influence by feed-forward ECs on vestibular sensory feedback signals might be a general property of vertebrate gaze control during locomotion (Figure 6).

## Conclusion

Maintaining visual world stability during locomotion is a major challenge for all vertebrates. Avoiding retinal image slip due to passive or self-generated head/body motion is traditionally ascribed to the transformation of mechanosensory feedback signals into ocular motor commands that offset perturbing head movements to ensure stable eye position in space. However, this generally acknowledged concept has been challenged over the past decade by increasing evidence that ascending neuronal pathways enable the propulsive CPG circuitry in the spinal cord to directly access and drive the brainstem ocular motor system during locomotion. Various types of *in vitro* experiments on larval and young adult *Xenopus laevis* provided the initial demonstration that efference copies of axial- as well as limb-based locomotor commands are able to elicit retinal image-stabilizing eye movements. This intrinsic feed-forward mechanism is spatio-temporally specific, functionally appropriate and dynamically adaptive, being capable of initiating gaze-stabilizing eye movements according not only to the immediate strength of ongoing locomotion but also to its mode, even throughout the transitional period of metamorphic development as one locomotor strategy progressively replaces the other. The ability to provide retinal image stability during the change in body format, propulsive motion profile and associated visual demands derives from a remarkable rewiring plasticity of spino-ocular motor coupling pathways that co-exist in the metamorphosing animal. While the impact of predictive locomotor efference copies matches the specificity of reactive sensory-motor transformations, the two fundamentally different signals do not simply summate in the production of image-stabilizing ocular motor commands. Rather, depending on the intensity of spinal CPG activity, the horizontal VOR is selectively suppressed, at least in part by an attenuation of the motion signal encoding at the sensory periphery in the inner ear. Significantly, this gaze-stabilizing mechanism that relies on locomotor EC signaling is not merely idiosyncratic to amphibians with their simpler and more stereotyped locomotor movement profiles, but evidently is also employed by other vertebrates, including humans. Recent studies in both *Xenopus* and mice (França de Barros et al., 2021; Bacqué-Cazenave et al., 2022) suggest that locomotor ECs can be used differently across species, depending on the biomechanical properties of their propulsive systems, their body organization, and the complexity of the sensory reference frames used to detect effective motion in space (Figure 6). This mechanism therefore represents an appealing example of a fundamental neural computation process that emerged early during vertebrate evolution and which was subsequently preserved, although necessarily superimposed by additional integrative mechanisms that accompanied the phylogenetic increase in sensory and motor system complexity.

## Author contributions

HS and JS wrote the first draft of the manuscript. FL made the figures. HS, FL, and JS reviewed and edited the final version of the manuscript. All authors contributed to the article and approved the submitted version.
